# Omega-3 eicosapentaenoic polar-lipid rich extract from microalgae *Nannochloropsis* decreases plasma triglycerides and cholesterol in a real-world normolipidemic supplement consumer population

**DOI:** 10.3389/fnut.2024.1293909

**Published:** 2024-02-06

**Authors:** Eneko Ganuza, Eghogho H. Etomi, Magdalena Olson, Corrie M. Whisner

**Affiliations:** ^1^Qualitas Health Inc., Houston, TX, United States; ^2^Auka Biotech SL., Iruña/Pamplona, Spain; ^3^College of Health Solutions, Arizona State University, Phoenix, AZ, United States

**Keywords:** polar lipids, cardiovascular health, cholesterol, triglycerides, microalgae, *Nannochloropsis*, omega-3 eicosapentaenoic acid (EPA), dietary supplements

## Abstract

**Introduction:**

AlmegaPL^®^ is an oil rich in polar-lipid (> 15% w/w) derived from the microalga *Nannochloropsis*, that contains exclusively eicosapentaenoic acid (EPA > 25% w/w), without the DHA that is present in all other natural sources of omega-3. Previous findings from a randomized controlled clinical trial demonstrated the ability of AlmegaPL^®^ supplementation to reduce cholesterol levels.

**Methods:**

In this post-market cohort study, we built upon previous findings and targeted the actual end-users of the supplement. Participants were recruited from a new subscriber database of AlmegaPL^®^ capsules (1000–1100 mg/day) to capture the complexity of real-world clinical and consumer settings. Changes in circulating triglycerides (TG), remnant cholesterol (RC), low-density lipoprotein cholesterol (LDL), high-density lipoprotein cholesterol (HDL), total cholesterol (TC), high-sensitivity C-reactive protein (hs-CRP), glucose and glycated hemoglobin (HbA1c) were monitored at baseline, Month 3, and Month 6 of supplementation using the at-home Baseline Heart Health Testing Kit by Imaware^®^ (Houston, TX, USA).

**Results:**

Participants, who had, on average, normal TG level at baseline (1.62 ± 0.60 mmol/L), experienced a significant and progressive decrease in TG at Month 3 (8.0%; −0.13 ± 0.59 mmol/L; *p* < 0.001) and Month 6 (14.2%; −0.23 ± 0.64 mmol/L; *p* < 0.001) (primary outcome). Furthermore, after 6 months of supplementation, TC and non-HDL-cholesterol decreased by 5.0% (−0.26 ± 0.98 mmol/L; *p* < 0.001) and 5.5% (−0.21 ± 0.86 mmol/L; *p* < 0.001) respectively, primarily driven by a 14.9% reduction in RC (−0.11 ± 0.29 mmol/L; *p* < 0.001).

**Discussion:**

Consistent with our previous clinical trial, the decrease in RC was not coupled to an increase in LDL, which seems to be a benefit associated with EPA-only based formulations. In addition, this study demonstrated the AlmegaPL^®^ capacity to maintain already healthy TG levels by further inducing a 14.9% decrease. Collectively, these findings highlight AlmegaPL^®^ uniqueness as a natural over-the-counter option for EPA-only polar lipid that appears particularly effective in maintaining blood lipid levels in a generally healthy, normolipidemic population.

**Clinical trial registration:**

https://clinicaltrials.gov/, identifier NCT05267301

## 1 Introduction

Cardiovascular disease (CVD) continues to be the leading cause of mortality globally, as reported by the World Health Organization (1). During the pandemic, CVD accounted for 1 in 5 fatalities in the USA, which was almost twice the fatalities attributed to COVID-19 (2). Inadequate intake of omega-3 polyunsaturated fatty acids (LCn-3 PUFA), specifically eicosapentaenoic acid (EPA; 20:5 n-3) and docosahexaenoic acid (DHA; 22:6 n-3), is estimated to contribute to approximately half of all CVD-related deaths annually (3). Surprisingly, 95% of the USA population fails to consume enough DHA and EPA (4), emphasizing the importance of achieving the recommended daily intake of LCn-3 PUFA (5) to support cardiovascular health.

The connection between LCn-3 PUFA intake and CVD was first proposed in a seminal study conducted in 1971, focusing on the Greenland Inuit population (6). Since that groundbreaking publication, over 4,000 clinical trials have been conducted on this topic, indicating the extensive research interest in this area (7). Notably, the pharmaceutical industry has recently undertaken large-scale clinical trials, encompassing much larger sample sizes compared to studies conducted in the preceding five decades, with emphasis on key disease endpoints such as major adverse cardiovascular events (MACE) (7). Traditionally, LCn-3 PUFA clinical research predominantly utilized fish oil and other complex sources containing both EPA and DHA. However, some of the recent pharmaceutical trials specifically investigated EPA-only treatments (JELIS, REDUCE-IT, RESPECT-EPA) and demonstrated superior outcomes compared to trials using combined DHA and EPA treatments (VITAL, ASCEND, STRENGTH, OMEMI). This finding has ignited a significant debate regarding the distinct roles of each LCn-3 PUFA in cardiovascular protection, with EPA apparently emerging as a prominent player in this regard (8–10).

The microalgae *Nannochloropsis* produces exclusively EPA, unlike all other natural sources of LCn-3 PUFA (e.g., fish, krill, heterotrophic microalgal oils) that also contain DHA. Besides EPA, this photoautotrophic organism also produces many other bioactive molecules such as pigments and phytosterols that could provide further cardiovascular benefit (11). AlmegaPL^®^ containing 25% w.w EPA is the first lipid extract derived from the *Nannochloropsis* made available in 2014 for human consumption (12). Besides being the only over-the-counter source of EPA-only, AlmegaPL^®^ also includes 15% w/w of polar lipids with a distinctive profile (galactolipids, phospholipids, and sulfoquinovosyldiacylglycerol) that provides functional characteristics distinct from other forms of LCn-3 PUFAs (such as triacylglycerides, phospholipids and ethyl esters). These unique polar lipid properties confer surfactant properties that promote the spontaneous formation of micelles in the digestive tract, facilitating the digestion and delivery of LCn-3 PUFA while minimizing undesirable fishy burps and aftertaste. Studies have shown that LCn-3 PUFA in AlmegaPL^®^ exhibit superior bioavailability compared to other forms of LCn-3 PUFA (13).

The cardioprotective benefits of AlmegaPL^®^ were initially demonstrated in a randomized, double-blinded clinical trial, where it led to significant decreases in remnant cholesterol (RC) (25%; *p* = 0.002) and total cholesterol (TC) (5%; *p* = 0.012) compared to placebo (14). Typically, the decrease in atherogenic lipid levels induced by LCn-3 PUFA is driven by the reduction in triglycerides (TG) secretion by the liver, often signaled by the decrease in RC (15). These markers are considered independent causal risk factors for MACE (16, 17) and might even have a better predictive value than low-density lipoprotein cholesterol (LDL) (18).

The relationship between RC reduction and TC has been a subject of controversy, but recent reports suggest that it may depend on the type of LCn-3 PUFA used (19). While both DHA and EPA can decrease RC, formulations containing DHA have shown an increase in LDL levels in response to the RC decrease (19–21). In contrast, EPA-only formulations have been associated with a reduction in RC without an increase in LDL (22), a mechanism of action also observed with AlmegaPL^®^ (14). Thus, LCn-3 PUFA supplementation affects blood lipids by decreasing TG, RC, and, in the case of EPA-only formulations, TC, all of which are associated with cardiovascular health (19). This is important because it implies that LCn-3 PUFA, particularly EPA-only formulations, are complementary to lipid lowering agents that primarily target LDL. Statins and phytosterols are successful at decreasing LDL, but even when LDL has been effectively controlled, there is substantial residual CVD risk associated to high TG and RC (8), which has been the focused of several pharma clinical trials (23). Therefore, in this dietary supplement trial TG was selected as the primary outcome because of the persistent risks present even in a generally healthy population (16).

Given the unique composition and positive results observed in our previous clinical trial conducted under controlled conditions, the aim of the present post-market cohort study was to confirm the cardiometabolic benefits of AlmegaPL^®^ in real-world clinical and costumer settings. By recruiting free-living adults who are consumers of this supplement, we sought to better reflect the complex conditions in which this supplement is intended to be effective.

## 2 Materials and methods

### 2.1 Clinical trial design, registration, and ethical approval

This clinical trial adhered to the International Conference on Harmonization (ICH) Guideline for Good Clinical Practice (GCP), the Notice for Guidance on Good Clinical Practice, and the Additional Ethical Considerations Guidelines. The trial received approval from the Argus Independent Review Board Committee (Tucson, Arizona) and is registered on the Clinicaltrials.gov Registry (NCT05267301).

This study followed an open-label, single arm design with a 6 month supplementation monitoring period. Its objective was to assess the effect of AlmegaPL^®^ on cardio-metabolic parameters and inflammatory markers in men and women. This study was conducted between May and November 2022, with participants located across the USA.

### 2.2 Participants

Potential participants were recruited through email invitations using the new-subscriber database from the AlmegaPL^®^ commercial website (24). Following a preliminary screening via the email questionnaire, participants were enrolled in the trial after providing written-informed consent.

A total of 480 otherwise healthy male and female volunteers over 18 years of age were enrolled from various regions in the USA. Exclusion criteria included unstable or serious illness (including but not limited to kidney, liver, and gastrointestinal disease, MACE, or diabetes), malignancy or treatment for malignancy within the previous 2 years, and allergic reactions to any of the supplement ingredients. Consequently, all participants were recruited regardless of their blood lipid levels.

### 2.3 Investigational product

The investigational product, supplied by Qualitas Health (Houston, TX, USA) under the brand name iwi, a vegetarian capsule containing 1000–1100 mg AlmegaPL^®^ . AlmegaPL^®^ is a lipid ethanol extract derived from whole-cell *Nannochloropsis oculata* QH5, a photoautotrophic microalga privately deposited at the University of Texas at Austin (UTEX) Culture Collection of Algae, which is rich in EPA conjugated to galactolipids and phospholipids. This marine microalga was grown in open pond raceways in Columbus, New Mexico and Imperial, Texas using brackish water and non-arable land. Participants were asked to consume a single capsule per day. Each capsule was standardized to contain a minimum of 1000 mg AlmegaPL^®^, which provided at least 250 mg EPA, 150 mg of polar lipids, 40 mg of arachidonic acid (ARA; 20:45 n-6), and 90 mg of palmitoleic acid (16:1 n-7). Additionally, it contained 23 mg of phytosterols and 15 mg of chlorophyll, 764 μg lutein, 387 μg zeaxanthin, and 541 μg beta-carotene analyzed according to Eurofins methods (Des Moines, IA, USA). The product is registered for use as a new dietary ingredient (NDIN) in the USA (12).

### 2.4 Intervention and study procedure

Upon enrollment, participants who provided signed consent forms were instructed to complete the Baseline Heart Health Testing Kit from Imaware^®^ (Houston, TX, USA) before consuming the supplement. Participants performed finger prick blood sampling after an 8-h fast, and the resulting dried blood spot samples were sent to a CLIA/CAP certified laboratory for analysis using the return shipping label in the test kit. The aggregated results were then shared by Imaware via a secure HIPAA and SOC2 API in a protected data environment. Once baseline measures were obtained, participants were instructed to orally take 1000–1100 mg of encapsulated AlmegaPL^®^ per day. This dosing regime was selected based on current standard dosing guidelines for the investigational product. Participants were then required to repeat this process after 3 months (mid-point) and 6 months (completion) of supplementation to monitor the progress from baseline.

Participants were advised to maintain their usual level of physical activity and diet throughout the study. Email questionnaires were used to assess any changes in activity level (exercising at least 150 min per week) and/or diet (2 servings of seafood intake/week, LCn-3 PUFA supplementation), which were considered during result analyses at each data point of the study. Compliance with the supplementation protocol was evaluated at the end of the study by counting the number of capsules used and determining the remaining product in the container. Participants with more than 20% of the assigned capsules remaining were considered non-compliant. Additionally, the authors monitored the participants by email or phone for any potential adverse reactions and adherence to the protocol at each data collection timepoint.

### 2.5 Outcome measures

Triglycerides (TG) (primary outcome), total cholesterol (TC), low-density lipoprotein cholesterol (LDL), high-density lipoprotein cholesterol (HDL), glycated hemoglobin (HbA1c), high-sensitivity C-reactive protein (hs-CRP), and glucose were analyzed using reagents for a clinical chemistry analyzer (Cobas 6000 and Cobas 8000, Roche Diagnostics, Indianapolis, IN). Remnant cholesterol (RC; calculates estimated following the VLDL calculation; VLDL = TC - LDL - HDL), non-high-density lipoprotein cholesterol (non-HDL-C; calculated as TC - HDL), and TG/HDL ratio (in mg/dL) were all calculated. Anthropometric measurements (height, weight, and body mass index) and lifestyle information (diet including seafood intake/week, LCn-3 PUFA supplementation, exercise, smoking, alcohol, and caffeine intake) were collected via email questionnaire. Participant safety was assessed by recording adverse events (AE) and serious adverse events (SAE). Protocol adherence was monitored through emailed and phone questionnaires at each data collection timepoint.

### 2.6 Statistical analyses

A power and sample size calculation were performed on the primary outcome measure (TG) using G*Power 3.1 (Department of Psychology, University of Düsseldorf, Germany). Based on a two-tailed student *t*-test with an effect size of 0.5 and an allocation ratio N2/N1 of 1, the sample size was estimated to be 210 to achieve a power of 95%. Preliminary data helped us estimate a relatively high dropout rate of 65%, but financial compensation to decrease dropout was discharged to avoid influencing real-world consumer settings. Therefore, accounting for a 65% dropout rate, a total of 480 participants were enrolled, aiming to achieve a proposed power of >0.95 to detect a statistically significant difference between the two groups of data.

The outcome results were statistically analyzed using an intention-to-treat (ITT) approach, which included participants who followed the supplementation schedule and completed baseline and at least one additional test (*n* = 256). Additionally, the per protocol (PP) population was defined as participants who followed the AlmegaPL^®^ supplementation schedule, completed baseline, and at least the Month 6 test (*n* = 223). Before conducting the analyses, all outcome data were checked for normality using the Kolmogorov-Smirnov Test and the Shapiro-Wilk Test. As the data did not follow a normal distribution, the non-parametric Wilcoxon Ranks sum test was used to compare two-tailed differences between baseline and either Month 3 or Month 6. To account for multiple variables, the thresholds for statistical significance of the 20 clinical outcomes were adjusted using the Bonferroni correction, resulting in a significance level of *p* < 0.0025.

*Post hoc* analyses were conducted to examine potential dropout bias associated with low baseline TG results. The Mann-Whitney test was used to compare two-tailed differences between the dropout population and the population that completed at least another test. Furthermore, dropout bias for underperforming participants was analyzed by comparing the TG delta at Month 3 between participants who also completed the Month 6 test and those who dropped out after completing the Month 3 test, using the Mann-Whitney Test. All statistical analyses were performed using SPSS Inc., Released in 2021, Version 28.0. Armonk, NY: IBM Corp.

## 3 Results

### 3.1 Participants

**FIGURE 1:**
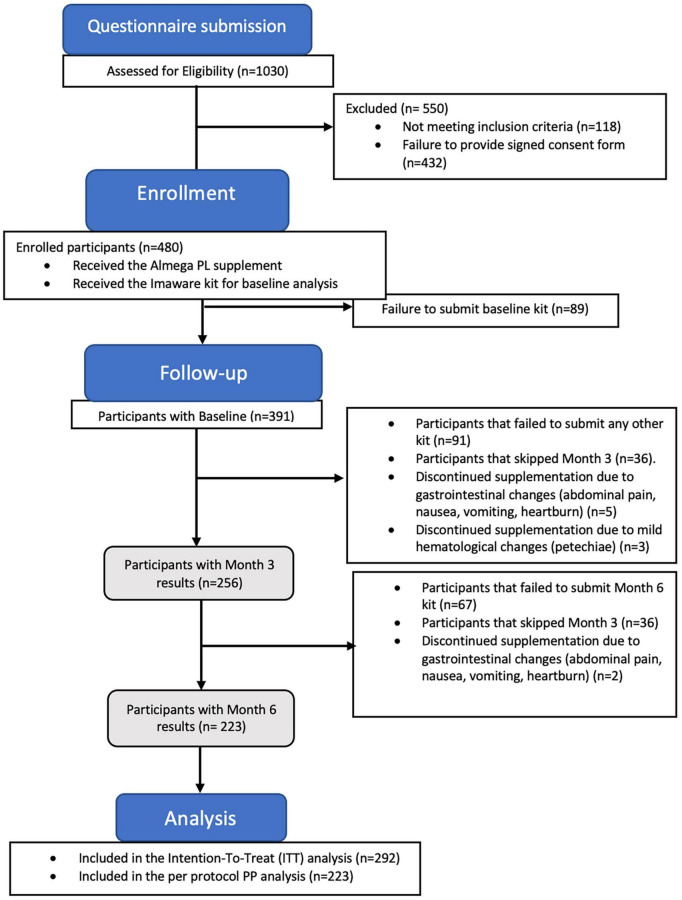
The study flow CONSORT diagram illustrates the number participants who completed the study after submitting the results at Month 6 (*n* = 223 per protocol population), along with the participants who completed at least 2 tests (*n* = 292 intention-to-treat population).

Of the initial 480 participants enrolled in the study, only 10 withdrawals were observed due to mild adverse effects, indicating a good tolerance to the supplement (Figure 1). Among these withdrawals, three participants reported increased bruising, including increased bleeding and petechiae, while seven participants reported abdominal discomfort, including symptoms such as abdominal pain, nausea, vomiting, and heartburn, following supplementation. Participants with adverse effect were advised to consult with their doctor. The vast majority of withdrawals were not related to adverse effects due to supplementation, but were primarily due to issues with adherence to the supplementation schedule, problems with the at-home testing user guide, cancelation of subscription for supplementation, or medical advice to discontinue all supplements due to an intervention or initiation of a new treatment plan which ultimately resulted in a failure to submit baseline (*n* = 89), Month 3 (*n* = 91), and Month 6 test kits (*n* = 67).

The all-baseline (*n* = 391) and ITT population (*n* = 292) were predominantly female in their early 50s, non-smokers and moderate alcohol consumers, who exercise frequently (Table 1). The ITT population was slightly overweight (BMI = 26.6 kg/m^2^). There were no significant changes in exercise, diet, and other lifestyle parameters from baseline through 6 months of supplementation. The lifestyle changes included aerobic activity (not less than 150 min/week), smoking habits, seafood intake, intake of other sources of omega- 3, excess alcohol, or caffeine intake. Baseline lipid assessment of the cohort confirmed that AlmegaPL^®^ consumers were normolipidemic (Table 2), with average TG (1.62 mmol/L), TC (5.14 mmol/L), and LDL (3.04 mmol/L) bellow their respective borderline limits (1.70, 5.17, and 3.36 mmol/L, respectively) (25). The supplementation compliance success rate was 81% among the ITT population.

**TABLE 1:** Baseline anthropometric and lifestyle parameters of participants.

Parameter	All baseline (*n* = 391)	ITT population (*n* = 292)
Female (*n*)	265	199
Male (*n)*	126	93
Age (years)	50 ± 10	51 ± 10
Weight (kg)	76 ± 18	78 ± 19
Height (m)	1.7 ± 0.1	1.7 ± 0.1
BMI (kg/m^2^)	26.6 ± 5.2	27.1 ± 6.2
Smokers (%)	1.3	0.7
CVD diagnosed excluding MACE (%)	31.8	32.3
CVD family history (%)	51.6	50.0
Diabetes diagnosed (%)	8.8	7.8
Diabetes family history (%)	38.8	35.8
Use of fish oil or LCn-3 PUFA supplementation (%)	36.6	39.0
Use of Statin or LCn-3 PUFA containing medication (%)	11.8	12.3
Use of other cholesterol or TG lowering supplement (%)	12.8	13.7
Exercising at least 150 min per week (%)	65.7	66.1
At least 2 seafood servings/week (%)	33.0	32.5
At least 14 alcoholic drinks/week (%)	3.8	4.5
At least 4 caffeinated drinks/day (%)	4.6	5.5

CVD, cardiovascular disease; MACE, major adverse cardiovascular events. Values represented as mean ± SD. No significant differences were observed between the All Baseline group and the intention-to-treat ITT population (*p* < 0.05).

**TABLE 2:** The effect of AlmegaPL^®^ supplementation for 6 months on cardiometabolic and inflammatory markers for the intention-to-treat (ITT) (*n* = 292) and per protocol (PP) population (*n* = 223).

	Baseline	Month 3	Δ 0M–3M	Month 6	Δ 0M–6M
Number of participants	**292**	**256**	**256**	**223**	**223**
TG	1.62 ± 0.60	1.48 ± 0.55*	**−**0.13 ± 0.59	1.39 ± 0.53*	**−**0.23 ± 0.64
TC	5.14 ± 0.91	5.02 ± 0.93	**−**0.12 ± 1.02	4.92 ± 0.95*	**−**0.26 ± 0.98
RC	0.74 ± 0.28	0.68 ± 0.25*	**−**0.06 ± 0.27	0.64 ± 0.24*	**−**0.11 ± 0.29
LDL	3.04 ± 0.78	2.99 ± 0.75	**−**0.04 ± 0.81	2.94 ± 0.78	**−**0.12 ± 0.77
HDL	1.37 ± 0.34	1.35 ± 0.34	**−**0.02 ± 0.30	1.34 ± 0.35	**−**0.05 ± 0.31
Non-HDL-C	3.78 ± 0.81	3.67 ± 0.79	**−**0.10 ± 0.87	3.58 ± 0.86*	**−**0.21 ± 0.86
TG:HDL ratio	2.94 ± 1.50	2.70 ± 1.34	**−**0.20 ± 1.24	2.60 ± 1.33*	**−**0.33 ± 1.30
hs-CRP	2.84 ± 5.60	1.98 ± 2.43	**−**0.67 ± 4.98	2.13 ± 1.84	**−**0.92 ± 5.85
GLU	4.57 ± 1.22	4.52 ± 0.93	**−**0.07 ± 1.46	4.79 ± 1.10	0.21 ± 1.46
HbA1c	5.58 ± 0.60	5.56 ± 0.39	**−**0.02 ± 0.45	5.58 ± 0.44	0.01 ± 0.53

Values represented as mean ± SD, *significantly different from baseline, *p* < 0.0025, triglycerides (TG; mmol/L), total cholesterol (TC; mmol/L), remnant cholesterol (RC; mmol/L), low-density lipoprotein cholesterol (LDL; mmol/L), high-density lipoprotein cholesterol (HDL; mmol/L), non-HDL-cholesterol (non-HDL-C; mmol/L), TG:HDL ratio, high-sensitivity C-reactive protein (hs-CRP; mg/L), glucose in plasma (GLU; mmol/L), hemoglobin A1c (HbA1c;%), change between baseline and month 3 (Δ0M–3M), change between baseline and month 6 (Δ0M–6M).

### 3.2 Cardiometabolic markers

AlmegaPL^®^ supplementation for 6 months significantly decreased TG by 14.2% (**−**0.23 ± 0.64 mmol/L; *p* < 0.001), TC by 5.0% (**−**0.26 ± 0.98 mmol/L; *p* < 0.001), RC by 14.9% (**−**0.11 ± 0.29 mmol/L; *p* < 0.001), non-HDL-C by 5.5% (**−**0.21 ± 0.86 mmol/L; *p* < 0.001) and TG to HDL cholesterol ratio by 11.3% (**−**0.33 ± 1.30 mmol/L; *p* < 0.001) (Table 2). hs-CRP decreased by 25.3% after 3 months and 30.2% after 6 months from baseline, albeit the decrease at the end of the study was not statistically significant (**−**0.92 ± 5.85 mg/L; *p* = 0.146).

The baseline TG results of the dropout population (1.54 ± 0.61 mmol/L; *n* = 99) were not statistically significantly different (*p* = 0.234) from the population that completed at least one of the subsequent tests (1.62 ± 0.60 mmol/L; *n* = 292), suggesting that there was no dropout bias associated with low baseline TG levels. Likewise, the Month 3 TG change (Δ0M**–**3M) of participants who also completed Month 6 (**−**0.10 ± 0.56 mmol/L; *n* = 187) was not statistically significantly different (*p* = 0.442) from the Δ0M–3M of those participants who dropped out after completing the Month 3 blood sample (**−**0.22 ± 0.66 mmol/L; *n* = 69), suggesting we had no dropout bias for underperforming participants.

## 4 Discussion

The participants recruited in the present post-market cohort study are reflective of the health-conscious consumers of this supplement, with the majority being non-smokers (99%), moderate alcohol consumers (96.2%), predominantly female (67%), in their early 50s, marginally overweight (BMI = 26.6 kg/m2), but active (65.7%) (Table 1). The mean baseline TG (1.62 ± 0.60 mmol/L) and TC (5.14 ± 0.91 mmol/L) concentrations were within the normal range according to the Adult Treatment Panel Guidelines (ATP III) (25), confirming the overall health of the real-world population that consumed the AlmegaPL^®^ supplement. Although the population participating in this study represented a normolipidemic population (only 12.3% of the population was pharmaceutically medicated for dyslipidemia), it’s important to note that lower TG levels, even within the normal range < 1.7 mmol/L), are still associated with better cardiovascular health (16). For this reason, the ability of AlmegaPL^®^ supplementation to significantly reduce TG levels already in the normal range offers valuable cardiometabolic support for healthy, active adults. Precisely, the population participating in this study represents a broad non-diseased target for primordial CVD prevention that could improve their cardiovascular health through over-the-counter dietary supplementation, prior to reaching clinically-indicated levels requiring pharmaceutical intervention (25).

The daily intake of LCn-3 PUFA in AlmegaPL^®^ (250 mg/day), while consistent with the promotion of cardiovascular health in a generally healthy population (26), is well below the intake levels (2000–4000 mg/day) recommended to treat CVD in diseased participants (23). Thus, AlmegaPL^®^ is intended for healthy adults seeking cardiometabolic support rather than for treatment of CVD. The impact of LCn-3 PUFA intake on the TG levels follows a dose-response relationship, even at relatively low supplementation levels (200–500 mg/day), where the TG decrease is estimated at 3.1–7.2% relative to baseline (27). In the present study, AlmegaPL^®^ decreased TG levels by 14.2% (**−**0.23 ± 0.64 mmol/L; *p* < 0.001) after 6 months (primary outcome) of supplementation, a response equivalent to 4-times the DHA + EPA dose reported for other LCn-3 PUFA sources (27). This suggests that LCn-3 PUFAs alone might not explain the high response obtained with AlmegaPL^®^, and some other factors might be at play. While EPA-only composition outperformed DHA + EPA in terms of decreasing MACE (8), there is no consensus on whether the EPA-only composition truly increases the LC-3 PUFA capacity to lower TG (28, 29). In turn, the polar form (glycolipids and phospholipids) to which the EPA is conjugated in AlmegaPL^®^ may partially explain the greater reductions observed for TG. Polar lipids extracted from soybean have been shown to decrease both TG and TC independently from their fatty acid profile (30), suggesting AlmegaPL^®^ polar lipids may also contribute to the observed lipid-lowering effect. Furthermore, minor constituents of the algal oil, such as phytosterols (31) and palmitoleic acid (32), may reinforce the observed lipid homeostasis.

Participants, who had, on average, normal TG level at baseline (1.62 ± 0.60 mmol/L), experienced a significant and progressive decrease in TG at Month 3 (8.0%; **−**0.13 ± 0.59 mmol/L; *p* < 0.001) and Month 6 (14.2%; **−**0.23 ± 0.64 mmol/L; *p* < 0.001) (primary outcome). The magnitude of TG lowering is influenced by the baseline TG level, with lower baseline TG levels associated with lower reductions (19). This illustrates the difficulty of demonstrating supplementation efficacy in an already healthy population compared to a population with dyslipidaemia, a condition with more room for improvement. The baseline TG level (1.6 ± 0.60 mmol/L) in the present post-market cohort study is by design more reflective of the real-world population taking this supplement than in the previous clinical trial (1.0 ± 0.59 mmol/L) (14). While both studies targeted a normolipidemic population (TG < 1.7 mmol/L), the baseline TG of the clinically standardized population selected previously was surprisingly low, which explains why AlmegaPL^®^ supplementation did not significantly decrease TG in that trial (14).

RC is the cholesterol content of triglyceride-rich lipoproteins, which correlates closely with plasma TG, as these lipoproteins are predominantly responsible for TG transport. The liver responds to LCn-3 PUFA supplementation by increasing TG oxidation, which contributes to the decrease in RC (15). This trend was observed in the present study, where, along with abovementioned TG, RC also decreased by 14.9% (**−**0.11 ± 0.29 mmol/L; *p* < 0.001) after 6 months of AlmegaPL^®^ supplementation. Consistent with our previous study (14) and with EPA-only formulations in general, LDL remains unchanged, ultimately resulting in significant decreases in TC (5.0%; **−**0.26 ± 0.98 mmol/L; *p* < 0.001) and non-HDL-C (5.5%), both of which are well-characterized risk factors for CVD. According to Varvo (33), RC, but not LDL, causes low grade inflammation, which explains why the inflammatory marker hs-CRP (high-sensitivity C-reactive protein) decreased by 30.2% (**−**0.92 ± 5.85 mg/L), albeit not significantly (*p* = 0.146). The lack of significance might be explained by the non-specific nature of the acute-phase hs-CRP response. This response can be dramatically triggered by many disorders unrelated to cardiovascular disease (e.g., infection) and may interfere with the interpretation of results in numerous CVD studies (34).

This is the second clinical trial that supports the use of AlmegaPL^®^ to promote cardiovascular health in a generally healthy adult population. The previous clinical trial (12), a randomized, placebo-controlled study, demonstrated in a controlled clinical setting, that AlmegaPL^®^ supplementation for 3 months significantly decreased TC, RC, and non-HDL-C compared to placebo. While placebo-controlled studies are considered the gold standard design for assessing the efficacy of new drugs and ingredients, they may create standardized conditions that deviate from the real-world complexity of supplementation use. These discrepancies in patient selection or treatment conditions may alter effectiveness of the supplement in the end users. Consequently, this second clinical trial was designed as a post-market cohort study that targeted actual consumers of this dietary supplement, accounting for the complexity associated with real-world clinical and consumer settings. The supplementation period was doubled from three (previous trial) to 6 months, and sample size increased from 120 to 480 participants. This second trial reinforced the previously observed decrease in TC, RL, and non-HDL-C, and also demonstrated a remarkable (14.2%) decrease in TG. In line with the dietary supplement’s health-supporting role, major disease hard endpoints were not measured. Despite these limitations, both studies observed the same mechanisms intrinsic to EPA-only drugs. This is important because, thus far, large pharmaceutical trials evaluating the MACE risk of mixed (DHA-containing) LCn-3 PUFAs have not shown the benefits of EPA ethyl esters, as seen in the REDUCE-IT and the JELIS trials (10, 21). Therefore, as EPA is emerging as the leading LCn-3 PUFA for the treatment of CVD in diseased patients, these clinical trials provide strong evidence for AlmegaPL^®^ supplementation to support cardiometabolic health in healthy adults by helping maintain blood lipids already within the normal range. AlmegaPL^®^ is the only natural source of EPA-only that is currently available over the counter for dietary supplementation. This novel ingredient provides a less processed and more affordable source of this fatty acid that fits the needs of the general population. In conclusion, AlmegaPL^®^ provides a natural EPA-only supplementation option, previously unavailable, to help maintain cardiovascular health in the general population.

## Data availability statement

The raw data supporting the conclusions of this article will be made available by the authors, without undue reservation.

## Ethics statement

The studies involving humans were approved by the Argus Independent Review Board Committee (Tucson, Arizona). The studies were conducted in accordance with the local legislation and institutional requirements. The participants provided their written informed consent to participate in this study. Written informed consent was obtained from the individual(s) for the publication of any potentially identifiable images or data included in this article.

## Author contributions

EG: Conceptualization, Data curation, Formal analysis, Funding acquisition, Investigation, Methodology, Project administration, Supervision, Validation, Visualization, Writing – original draft, Writing – review and editing. EE: Data curation, Formal analysis, Investigation, Methodology, Writing – review and editing. MO: Data curation, Formal analysis, Investigation, Methodology, Writing – review and editing. CW: Formal analysis, Investigation, Methodology, Writing – review and editing.
